# Foliar folic acid application enhances nitrogen assimilation and improves quality-related metabolites accumulation in *Dendrobium officinale*

**DOI:** 10.3389/fpls.2026.1918138

**Published:** 2026-09-14

**Authors:** Hongyan Meng, Mingqiong Zheng, Xiaobing Cheng, Wenhua Wang, Enming He

**Affiliations:** Xiamen Key Laboratory of Plant Introduction & Quarantine and Plant Product, Fujian Key Laboratory of Subtropical Plant Physiology and Biochemistry, Fujian Institute of Subtropical Botany, Xiamen, Fujian, China

**Keywords:** alkaloids, *Dendrobium officinale*, folate, foliar application, metabolomics, nitrogen assimilation, quality improvement

## Abstract

Folates are essential cofactors in one-carbon metabolism, yet their roles in coordinating nitrogen assimilation and the accumulation of quality-related metabolites in medicinal orchids remain poorly understood. Here, we profiled folate derivatives across tissues and stem developmental stages of *Dendrobium officinale* and evaluated how foliar nutrient treatments affect folate accumulation and nitrogen metabolism. Folate profiling revealed distinct tissue-specific patterns: leaves were the major sink for total and reduced folates, whereas flowers preferentially accumulated formylated folates. Foliar application of balanced nutrient solutions, particularly Huaduoduo No. 1, markedly increased total folate levels. *In vitro* enzyme assays showed that folates, especially 5-formyltetrahydrofolate (5-F-THF), enhanced nitrate reductase (NR) and nitrite reductase (NiR) activities, with a stronger effect on NiR. Consistent with these findings, foliar application of folic acid (FA; 15 mg L^-1^) increased NR and NiR activities *in planta*, accompanied by higher nitrate and lower nitrite levels. Untargeted metabolomic analysis further showed that FA treatment reprogrammed amino acid and alkaloid metabolism, increasing the abundance of tryptophan-derived metabolites and several bioactive alkaloids. Molecular docking further predicted favorable interactions between folates and the catalytic pockets of nitrogen metabolism-related enzymes, supporting a potential direct role of folates in regulating nitrogen assimilation. Collectively, these findings indicate that folate-associated regulation of nitrogen assimilation is linked to metabolic reprogramming in *D. officinale*. This study provides a practical folate-based strategy for enhancing the nutritional and medicinal quality of this economically important orchid.

## 1 Introduction

*Dendrobium officinale* Kimura et Migo is a highly valued epiphytic orchid widely used in traditional Chinese medicine and functional foods because of its diverse bioactive constituents and health-promoting properties (Wu et al., 2023a; Zhang et al., 2023). Its stems are rich in polysaccharides, alkaloids, amino acids, and phenolic compounds, which contribute to its immunomodulatory, antioxidant, and anticancer activities (Zhong et al., 2022; Zou et al., 2024; Shi et al., 2025). Given its considerable medicinal and commercial value, *D. officinale* has become an important horticultural crop. However, the quality of cultivated materials varies considerably, largely because of differences in cultivation practices and nutrient management, hindering standardized production and quality improvement (Deng et al., 2024; Hu et al., 2024). Among mineral nutrients, nitrogen is a major determinant of plant growth, biomass accumulation, and the biosynthesis of quality-related metabolites in *D. officinale* (Ge et al., 2021; Fan et al., 2023; Wu et al., 2024; Yan et al., 2026).

Nitrogen assimilation is tightly integrated with carbon metabolism and secondary metabolite biosynthesis. In C3 plants, ammonium assimilation through the glutamine synthetase (GS)/glutamate synthase (GOGAT) pathway requires carbon skeletons and reducing equivalents generated by photosynthesis, whereas photorespiration further connects carbon flux with nitrogen recycling (Hanson and Roje, 2001; Nunes-Nesi et al., 2010; Han et al., 2024). In *D. officinale*, nitrogen availability affects amino acid metabolism, alkaloid accumulation, and polysaccharide biosynthesis, thereby influencing plant growth and medicinal quality (Chen et al., 2021; Zhang et al., 2024; Yin et al., 2026). However, the molecular mechanisms underlying nitrogen utilization in this species remain poorly understood, particularly the potential contribution of one-carbon metabolism.

Folate-mediated one-carbon metabolism supplies one-carbon units for nucleotide biosynthesis, amino acid interconversion, and methylation reactions and is increasingly recognized as a link between nitrogen and sulfur metabolism in plants (Hanson and Roje, 2001). Studies in *Arabidopsis thaliana* have shown that folate homeostasis is associated with amino acid metabolism, nitrate assimilation, and adaptive responses to nitrogen starvation, suggesting that folate status may influence nitrogen use efficiency and metabolic reprogramming (Li et al., 2021; Liao et al., 2022; Meng et al., 2025b). Folates may also connect nutrient status with gene regulation and developmental processes through their role in methylation reactions (García-Valencia et al., 2025). Therefore, these findings indicate that folates function not only as essential metabolic cofactors but also as potential regulators of nutrient responses and plant development (Li et al., 2021; Meng et al., 2025b). However, whether folate metabolism similarly links nitrogen utilization with the biosynthesis of quality-related metabolites in medicinal orchids remains largely unknown.

To date, the folate metabolic profile of *D. officinale* has not been systematically characterized. In particular, the tissue-specific distribution of folate derivatives, developmental changes in folate levels in stems, and responses of folate metabolism to foliar nutrient treatments remain unknown. It is also unclear whether folate metabolites regulate key nitrogen assimilation enzymes and thereby influence the accumulation of quality-related metabolites, including amino acids and alkaloids. Elucidating these relationships would advance our understanding of folate function in medicinal orchids and provide a basis for developing rational nutrient-management strategies to improve *D. officinale* quality.

In this study, we profiled folate derivatives in different tissues and developmental stages of *D. officinale* using UHPLC-MS/MS and evaluated the effects of foliar nutrient treatments on folate accumulation. We further examined whether exogenous folate application affects nitrogen assimilation by measuring the activities of key nitrogen-metabolism enzymes and endogenous nitrate and nitrite levels *in planta*. Untargeted metabolomics was then used to characterize the effects of folic acid (FA) treatment on amino acid and alkaloid profiles. Finally, molecular docking was performed to investigate potential interactions between folates and nitrogen-metabolism enzymes. Collectively, this study aimed to elucidate how folates coordinate nitrogen assimilation and the accumulation of quality-related metabolites in *D. officinale*, thereby providing a mechanistic basis and practical strategy for improving the nutritional and medicinal value of this economically important orchid crop.

## 2 Materials and methods

### 2.1 Plant materials

Plant materials of *D. officinale* used in this study were obtained from the Fujian Institute of Subtropical Botany (Xiamen, China) and comprised rooted tissue-cultured plantlets maintained in a culture room and acclimatized potted plants grown in a greenhouse for one, two, or three years (Meng et al., 2025a). Greenhouse temperature and relative humidity were maintained at 25 ± 2 °C and 65 ± 5%, respectively. For folate metabolic profiling, healthy leaves from one-year-old stems, flowers, and stems from one-, two-, and three-year-old potted plants were collected (**Figure 1**). Two-year-old potted plants, obtained by transplanting tissue-cultured plantlets into greenhouse pots and maintaining them for two years, were used for foliar-spraying experiments and stems were collected for folate profiling (**Figures 2**). Uniform leaves from tissue-cultured plants were used to assess nitrogen-metabolism enzyme activities, with FA and 5-formyltetrahydrofolate (5-F-THF) added directly to the enzyme reaction systems (**Figure 3**). Leaves from two-year-old potted plants treated with FA foliar-spraying were collected for enzyme activity assays (**Figures 4**; **Supplementary Figure 1**).

**Figure 1:**
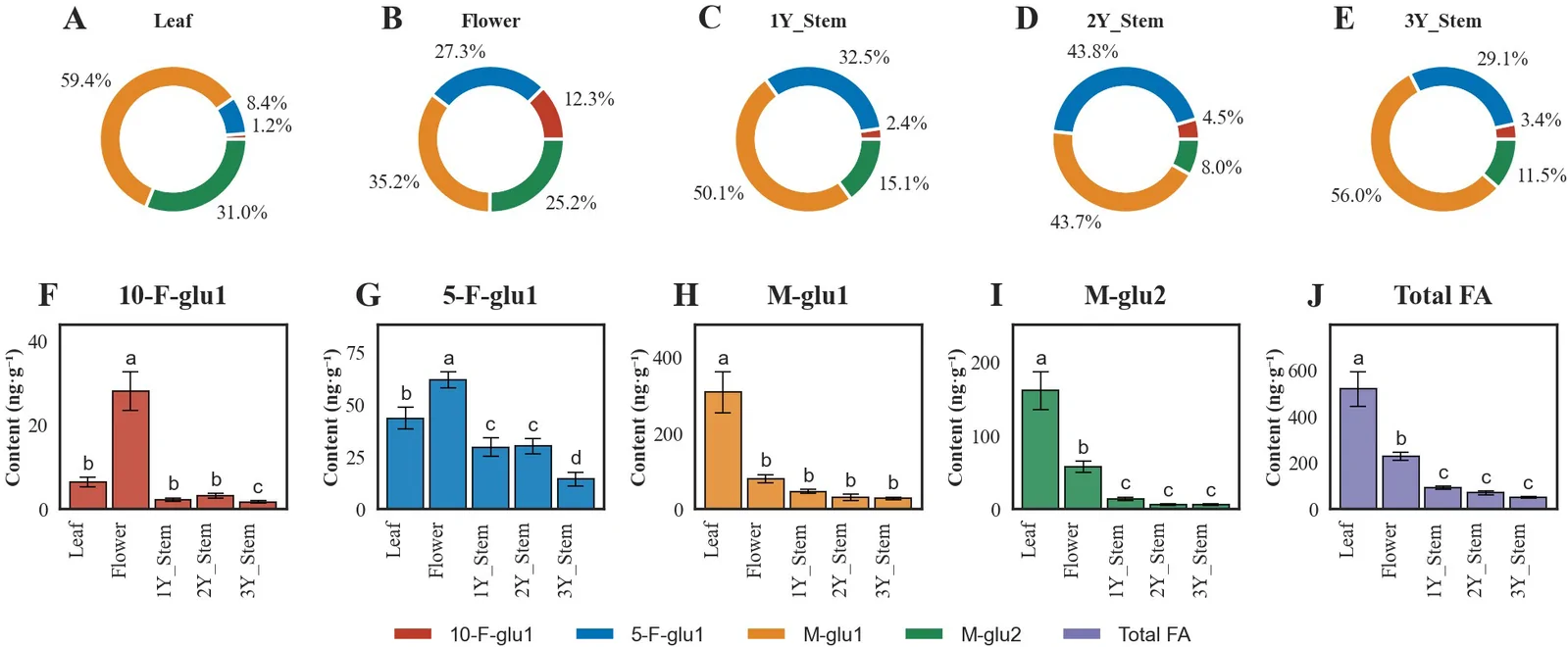
Folate profiles in different tissues and stems of different ages of *D. officinale*. **(A–E)** Relative composition of four folate derivatives in leaf **(A)**, flower **(B)**, 1-year-old stem (1Y_Stem, C), 2-year-old stem (2Y_Stem, D), and 3-year-old stem (3Y_Stem, E). **(F–J)** Absolute contents of 10-F-glu1 **(F)**, 5-F-glu1 **(G)**, M-glu1 **(H)**, M-glu2 **(I)**, and total folate **(J)** in different tissues and stems. 10-F represents 10-formyltetrahydrofolate; 5-F represents 5-formyltetrahydrofolate; M represents the total content of dihydrofolate, tetrahydrofolate, 5-methyltetrahydrofolate, 5,10-methylenetetrahydrofolate, and 5,10-methenyltetrahydrofolate; glux indicates the glutamic acid polymerization degree. Different lowercase letters above bars indicate significant differences at P < 0.05 (Tukey’s HSD test).

**Figure 2:**
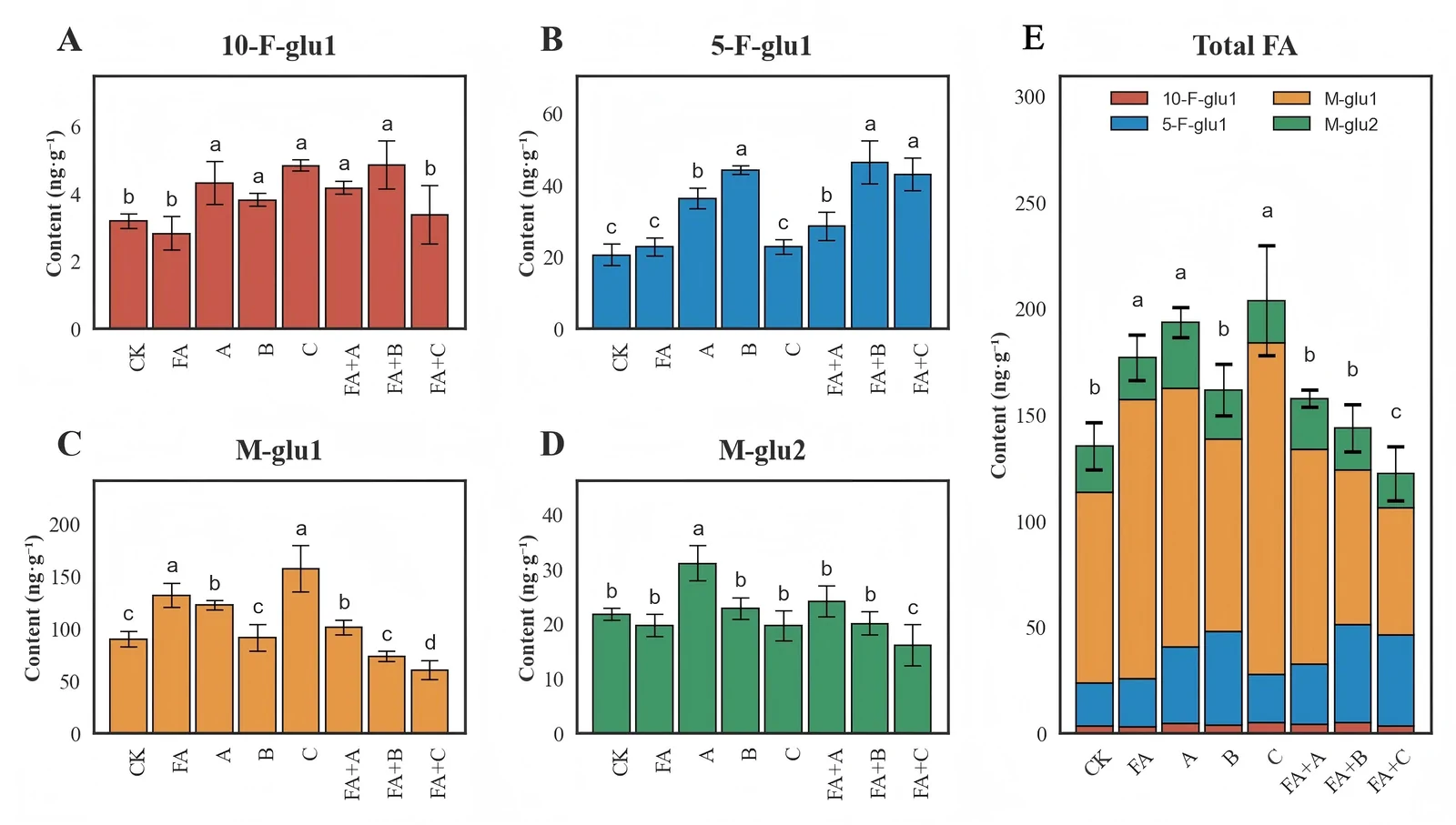
Effects of different foliar fertilizer treatments on the folate metabolic profile of *D. officinale*. **(A–E)** Absolute contents of 10-F-glu1 **(A)**, 5-F-glu1 **(B)**, M-glu1 **(C)**, M-glu2 **(D)**, and total folate **(E)** under different foliar fertilizer treatments. Different lowercase letters above bars indicate significant differences at P < 0.05 (Tukey’s HSD test). Treatments: CK, control; FA, 5 mg·L^-1^ folic acid; A, ManCui trace element type 20-10-20+TE; B, ManCui 20-10-20 + 0.5Ca+1Mg+TE; C, Huaduoduo No. 1; FA+A, FA+B, and FA+C, combinations of FA with A, B, and C, respectively. 10-F represents 10-formyltetrahydrofolate; 5-F represents 5-formyltetrahydrofolate; M represents the total content of dihydrofolate, tetrahydrofolate, 5-methyltetrahydrofolate, 5,10-methylenetetrahydrofolate, and 5,10-methenyltetrahydrofolate; glux indicates the glutamic acid polymerization degree.

**Figure 3:**
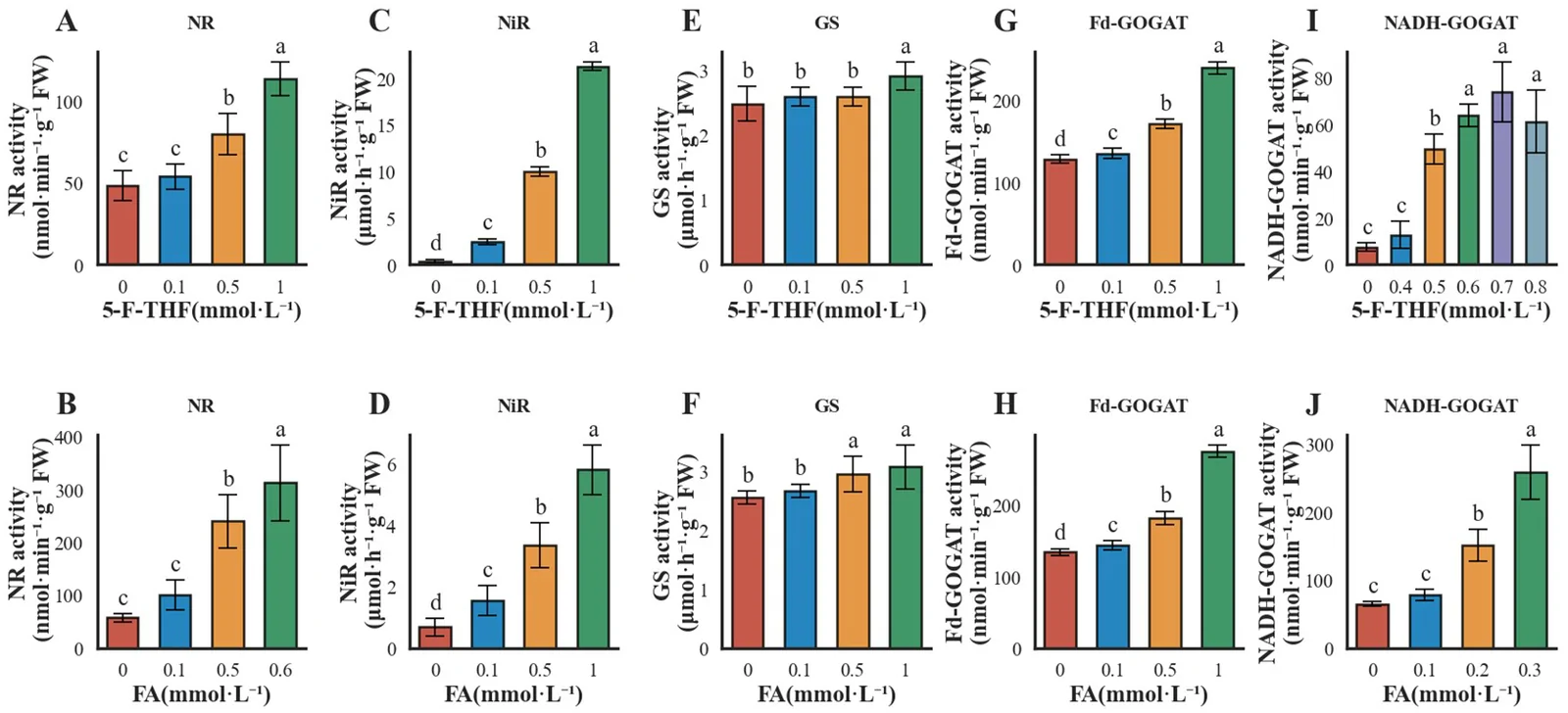
Effects of 5-F-THF and FA on the activities of nitrogen metabolism enzymes *in vitro*. **(A, B)** Nitrate reductase (NR) activity under different concentrations of 5-F-THF **(A)** and FA **(B)**, respectively. **(C, D)**. Nitrite reductase (NiR) activity under different concentrations of 5-F-THF **(C)** and FA **(D)**, respectively. **(E, F)** Glutamine synthetase (GS) activity under different concentrations of 5-F-THF **(E)** and FA **(F)**, respectively. **(G, H)** Ferredoxin-dependent glutamate synthase (Fd-GOGAT) activity under different concentrations of 5-F-THF **(G)** and FA **(H)**, respectively. **(I, J)** NADH-dependent glutamate synthase (NADH-GOGAT) activity under different concentrations of 5-F-THF **(I)** and FA **(J)**, respectively. Data are presented as mean ± SD (n = 12). Different lowercase letters above bars indicate significant differences among treatments at P < 0.05, as determined by Tukey’s multiple-comparison test.

### 2.2 Chemicals and reagents

Folate standards, including 5-F-THF (purity ≥95%) and FA (purity ≥98%), were purchased from Schircks Laboratories (Jona, Switzerland). Assay kits for nitrogen-metabolism enzymes and nitrogen-containing metabolites (nitrate-N, nitrite-N, ammonium-N, Glu, total amino acids, total protein and total alkaloids) were obtained from Suzhou Keming Biotechnology Co., Ltd. The principal assay kits and catalog numbers were as follows: nitrate reductase (NR, NR-1-Y), nitrite reductase (NiR, NIR-1-G), glutamine synthetase (GS, GS-1-W), NADH-dependent glutamate synthase (NADH-GOGAT, GOGAT-1-Y), ferredoxin-dependent glutamate synthase (Fd-GOGAT, GOGAT-1-W), nitrate-N (ZXTD-1-G), nitrite-N (SPYXY-1-G), ammonium-N (ZATD-1-G), Glu (GLU-1-Y), total amino acids (AA-1-W), total protein (BCAP-1-W), and total alkaloids (SWJ-2-Y). All solvents used for UPLC–MS/MS were chromatographic grade. Acetonitrile, methanol, and formic acid were purchased from Merck KGaA (Darmstadt, Germany), and 18.2 MΩ·cm deionized water was produced using a Milli-Q water-purification system (Millipore Corporation, Bedford, MA, USA). Three commercial foliar fertilizers were evaluated. Fertilizer A (ManCui trace element type 20-10-20+TE; Shandong Meishimeike Crop Nutrition Co., Ltd., Weifang, Shandong, China) consists of 20% N, 10% P_2_O_5_, 20% K, 0.3% MgO, 0.21% B, 0.03% Zn and 0.55% Arg. Fertilizer B (ManCui 20-10-20 + 0.5Ca+1Mg+TE, Shandong Meishimeike Crop Nutrition Co., Ltd., Weifang, Shandong, China) contained 20% N, 10% P_2_O_5_, 20% K, 0.5% Ca, 1.0% MgO, 0.05% B, and 0.03% Zn, as well as Thr, Glu, and proline-containing peptide proteins. Fertilizer C (Huaduoduo No. 1; Scotts Miracle-Gro Company, Marysville, OH, USA) was a 20-20-20+TE formulation containing 20% N, 20% P_2_O_5_, 20% K, 1.5% S, 0.12% Fe, 0.06% Mn, 0.02% B, 0.015% Zn, 0.7% Cu, and Mo. All fertilizers were diluted 1000-fold according to manufacturer’s instructions.

### 2.3 Foliar fertilizer treatments

Eight treatments were established: control (CK), 5 mg·L^-1^ FA, three commercial foliar fertilizers (A, B, and C), and three combined treatments (FA+A, FA+B, and FA+C). All fertilizers were diluted 1000-fold (1 g·L^-1^; 200 mg·L^-1^ N) and applied weekly for 16 weeks. Each treatment comprised four biological replicates, with five plants per replicate.

### 2.4 UPLC-MS/MS analysis of folate profiles

Sample preparation and metabolite extraction followed previously described methods with modifications (Sun et al., 2021; Meng et al., 2025b). Briefly, frozen plant tissues were homogenized in 50 mmol·L^–1^ sodium phosphate buffer (pH 7.4) containing 1% (w/v) sodium L-ascorbate and 0.1% (v/v) β-mercaptoethanol. After centrifugation, supernatants were purified using Captiva EMR-Lipid cartridges (Agilent Technologies) to remove lipids and other interfering compounds. Chromatographic separation was performed on a Poroshell 120 SB-C18 column (2.1 × 75 mm, 2.7 μm; Agilent) at 30 °C. The mobile phases were 0.1% formic acid in water (A) and acetonitrile (B), with the following gradient: 0–1 min, 1% B; 1–6 min, 1–20% B; 6–7 min, 20–30% B; 7–8 min, 30% B; and 8.1–10 min, 1% B. The flow rate was 0.3 mL·min^-1^, and the injection volume was 5 μL. Mass spectrometric detection was performed on an Agilent 6495 triple quadrupole mass spectrometer in positive electrospray ionization (ESI+) mode. Folate vitamers were quantified by selected reaction monitoring (SRM) of specific precursor-to-product ion transitions. Calibration curves were generated using authentic standards. Four biological replicates were analyzed per treatment.

### 2.5 Nitrogen metabolism enzyme assays

#### 2.5.1 *In vitro* enzyme activity assays

NR, NiR, GS, NADH-GOGAT, and Fd-GOGAT activities were measured using commercial assay kits (Suzhou Comin Biotechnology Co., Ltd., Suzhou, Jiangsu, China) according to the manufacturer’s protocols. Cell-free leaf extracts were prepared from tissue-cultured seedlings by grinding in liquid nitrogen. Reaction mixtures were incubated at 25 °C and pH 7.5 for 30 min and contained varying concentrations of 5-F-THF or FA (**Figure 3**). Enzyme activities were expressed as nmol·min^-1^·g^-1^ FW or µmol.h^–1^.g^–1^ FW. Each assay included four biological replicates and three technical replicates (n = 12).

#### 2.5.2 *In vivo* enzyme activity assays

Two-year-old potted plants were foliar-sprayed weekly with FA at 0, 5, or 15 mg·L^-1^ for 16 weeks. Leaves were collected 1 week after the final application, immediately frozen in liquid nitrogen, and stored at −80 °C until analysis. Enzyme activities were determined as described in Section 2.5.1. Four biological replicates were analyzed per treatment.

### 2.6 Quantification of nitrogen metabolites

Nitrate, nitrite, ammonium, Glu, total amino acids, total protein, and total alkaloids contents were determined using commercial assay kits (Suzhou Comin Biotechnology Co., Ltd., Suzhou, Jiangsu, China). For nitrate/nitrite/ammonium assays, 0.5 g fresh leaves were homogenized in 5 mL deionized water and centrifuged at 12,000 × *g* for 15 min at 4°C (Centrifuge 5424 R; Eppendorf SE, Hamburg, Germany); the supernatants were collected for analysis. Glu was extracted with 0.1 M HCl and quantified using a glutamate dehydrogenase-coupled colorimetric assay at 340 nm. Total amino acids were determined by the ninhydrin colorimetric method at 570 nm, using L-leucine as the standard. Total protein was extracted with RIPA lysis buffer containing 1 mM PMSF (Beyotime Biotechnology, Shanghai, China) and quantified by Coomassie Brilliant Blue G-250 staining at 595 nm, using bovine serum albumin (BSA) as the standard. Total alkaloids were extracted with 70% ethanol by ultrasonic treatment at 40 kHz for 30 min (KQ-500DE; Kunshan Ultrasonic Instruments Co., Ltd., Kunshan, Jiangsu, China) and quantified by the bromocresol green colorimetric method at 415 nm, using berberine hydrochloride as the standard. Absorbance was measured using a SpectraMax M5 microplate reader (Molecular Devices, LLC., San Jose, CA, USA). All data were expressed as mg·g^–1^ fresh weight (FW).

### 2.7 Widely targeted metabolomics

Based on preliminary enzyme activity results, 15 mg·L^–1^ FA was selected for metabolomic comparison with the control. For each treatment, three biological replicates were prepared, with each replicate comprising leaves pooled from three individual plants. Samples were freeze-dried, ground to a fine powder, extracted with pre-cooled 70% methanol containing internal standards, centrifuged, and filtered through 0.22-μm membranes before analysis by Wuhan MetWare Biotechnology Co., Ltd. (Wuhan, Hubei, China).

Metabolites were analyzed using an ExionLC™ AD UPLC system coupled to a QTRAP mass spectrometer (SCIEX, Framingham, MA, USA) equipped with an Agilent SB-C18 column (2.1 mm × 100 mm, 1.8 μm). The mobile phases were 0.1% formic acid in water (A) and 0.1% formic acid in acetonitrile (B), with the following gradient: 0–9 min, 5–95% B; 9–10 min, 95% B; 10–11.1 min, 95–5% B; and 11.1–14 min, 5% B. The flow rate was 0.35 mL·min^-1^, column temperature was 40 °C, and injection volume was 2 μL. Mass spectrometry was performed using ESI in both positive and negative modes at a source temperature of 550 °C and ion-spray voltages of 5500 V and −4500 V, respectively. Quantification was performed in multiple reaction monitoring (MRM) mode.

Raw data were processed using SCIEX OS for peak integration and correction. Principal component analysis (PCA) and orthogonal partial least-squares discriminant analysis (OPLS-DA) were performed using R packages; OPLS-DA models were validated with 200 permutation tests to assess overfitting. Differential metabolites were identified using a variable importance in projection (VIP) > 1 and absolute fold change (|Log2FC|) ≥ 1 by matching retention times, precursor ions, and MS/MS spectra against in-house and public databases, including MassBank, MoNA, and METLIN, followed by Kyoto Encyclopedia of Genes and Genomes (KEGG) annotation and metabolite set enrichment analysis.

### 2.8 Molecular docking simulations

The three-dimensional structures of nitrogen metabolism enzymes were retrieved from the AlphaFold Protein Structure Database (https://alphafold.ebi.ac.uk/; European Bioinformatics Institute, Hinxton, UK; accessed January 2025) (Fleming et al., 2025), including NR (*Arabidopsis thaliana*, UniProt ID: Q0WLH2, AlphaFold DB ID: AF-Q0WLH2-F1), NiR (*A. thaliana*, UniProt ID: Q39161, AF-Q39161-F1), GS (*A. thaliana*, UniProt ID: Q43127, AF-Q43127-F1), NADH-GOGAT (*A. thaliana*, UniProt ID: Q9LV03, AF-Q9LV03-F1), and Fd-GOGAT (*A. thaliana*, UniProt ID: Q9ZNZ7, AF-Q9ZNZ7-F1).

Ligand 3D structures of 5-F-THF (PubChem CID: 135403648) and FA (PubChem CID: 135398658) were obtained from PubChem (National Center for Biotechnology Information, Bethesda, MD, USA). Protein structures were prepared in PyMOL 3.0.3 (Schrödinger, LLC., New York, NY, USA) by removing water molecules and unrelated ligands and adding hydrogens. Ligands were energy-minimized using ChemDraw 20.0 (PerkinElmer, Inc., Waltham, MA, USA), and PDBQT files were generated with AutoDock Tools 1.5.7 (The Scripps Research Institute, La Jolla, CA, USA). Molecular docking was performed using AutoDock Vina 1.2.0 with grid boxes encompassing the entire protein (60 × 60 × 60 Å; grid spacing, 0.375 Å). Nine binding pockets were predicted for each enzyme, with one conformation generated per pocket. The conformation with the lowest binding energy was considered the optimal binding mode. Molecular interactions were visualized and analyzed using PyMOL 3.0.3 (Schrödinger, LLC., New York, NY, USA) and Discovery Studio 2021 (BIOVIA, San Diego, CA, USA). Rationale for using *Arabidopsis* protein structures: Because protein structures of *D. officinale* enzymes are unavailable in the AlphaFold database, *Arabidopsis* thaliana homologs were used as structurally relevant templates for molecular docking analysis.

### 2.9 Statistical analysis

Enzyme activity and nitrogen metabolism data were analyzed by one-way analysis of variance (ANOVA) followed by Tukey’s honestly significant difference (HSD) test using SPSS Statistics 26.0 (IBM Corporation, Armonk, NY, USA). Statistical significance was set at P < 0.05; different lowercase letters within a column indicate significant differences among treatments at P < 0.05. Data are presented as mean ± standard deviation. Figures were prepared using GraphPad Prism 9.5.0 (GraphPad Software, LLC., San Diego, CA, USA) and Adobe Illustrator 2024 (Adobe Inc., San Jose, CA, USA). Heatmaps were generated with the pheatmap package (version 1.0.12) in R 4.3.1 (R Foundation for Statistical Computing, Vienna, Austria).

## 3 Results

### 3.1 Folate metabolic profiles vary across tissues and stem ages in *D. officinale*

UPLC–MS/MS profiling revealed pronounced tissue-specific differences in folate derivative accumulation in *D. officinale* (**Figure 1**). In leaves, M-glu1 and M-glu2 (M-type reduced folates) were the predominant folate forms, followed by 5-F-glu1 and 10-F-glu1 (**Figure 1A**). Flowers showed a similar profile, although 5-F-glu1 was the second most abundant form (**Figure 1B**). The folate profiles of one- and three-year-old stems were also similar, with M-glu1 as the predominant derivative, followed by 5-F-glu1, M-glu2, and 10-F-glu1 (**Figures 1C, E**). However, in two-year-old stems, M-glu1 and 5-F-glu1 accumulated at similar levels and were the dominant forms, whereas M-glu2 and 10-F-glu1 occurred at lower concentrations (**Figure 1D**).

Quantitatively, the formylated derivatives 10-F-glu1 and 5-F-glu1 were most abundant in flowers, reaching 27.9 ± 4.6 ng·g^–1^ and 61.7 ± 3.8 ng·g^–1^, respectively, followed by leaves and stems (**Figures 1F, G**). In contrast, M-glu1 and M-glu2 accumulated predominantly in leaves, reaching 307.2 ± 53.5 ng·g^–1^ and 160.6 ± 25.4 ng·g^–1^, respectively, substantially exceeding their levels in flowers and stems (**Figures 1H, I**). Total folate content largely mirrored the distribution of M-type reduced folates, following the order leaves > flowers > stems (**Figure 1J**).

Collectively, these results demonstrate strong tissue-specific differences in folate metabolism in *D. officinale*. Leaves are the principal reservoir of total folates, particularly reduced folates, whereas flowers are relatively enriched in formylated folates. Stems contain comparatively low levels of folates, which tend to decrease with developmental age.

### 3.2 Differential regulation of folate metabolic profiles by foliar fertilizer treatments

To determine whether folate metabolism in *D. officinale* could be modulated by exogenous nutrient application, eight foliar treatments were evaluated. Folate derivatives exhibited distinct treatment-dependent responses. For 10-F-glu1, treatments A, B, C, FA+A, and FA+B significantly increased its accumulation relative to CK, with treatment C producing the highest level (4.8 ± 0.2 ng·g^-1^). In contrast, FA alone slightly decreased 10-F-glu1 relative to CK, whereas its combination with A or B increased 10-F-glu1 above the control level (**Figure 2A**). A similar treatment-dependent response was observed for 5-F-glu1. FA+B and FA+C significantly increased 5-F-glu1 accumulation, with FA+C producing a significantly higher level (42.7 ± 4.5 ng·g^-1^) than either FA alone (22.6 ± 2.5 ng·g^-1^) or C alone (22.6 ± 2.0 ng·g^–1^; **Figure 2B**). In contrast, M-glu1 and M-glu2 responded differently to foliar fertilization. Treatment C alone increased M-glu1 content 1.74-fold relative to CK, whereas FA+B and FA+C significantly reduced its accumulation. For M-glu2, treatment A produced the highest content (30.9 ± 3.2 ng·g^-1^), representing a 1.43-fold increase over CK, whereas FA+C reduced M-glu2 accumulation to 74% of the control level (**Figures 2C, D**).

Foliar fertilization also affected total folate accumulation. FA, A, and C all increased total folate content to varying degrees, with C producing the largest increase (203.4 ± 25.9 ng·g^-1^), followed by A (193.1 ± 7.2 ng·g^-1^). In contrast, combined treatments generally resulted in lower total folate levels than the corresponding single treatments, with FA+C producing the lowest content (122.0 ± 12.8 ng·g^–1^; **Figure 2E**). Thus, foliar treatments differentially affected individual folate derivatives and total folate accumulation in *D. officinale*, indicating that folate metabolism responds in a treatment-specific manner to exogenous nutrient supply.

### 3.3 Direct activation of nitrogen metabolism enzymes by 5-F-THF and FA *in vitro*

Because foliar application altered endogenous folate profiles, we next investigated whether folate compounds could directly modulate key enzymes involved in nitrogen metabolism using a cell-free system. To minimize the effects of cellular compartmentalization in intact plants, varying concentrations of 5-F-THF and FA were added directly to enzyme reaction mixtures to assess their immediate effects on enzyme activity.

Both 5-F-THF and FA affected the activities of nitrogen metabolism enzymes *in vitro* (**Figure 3**), with NR and NiR showing the strongest responses. FA more strongly activated NR than 5-F-THF, and its effect was concentration-dependent, with NR activity increasing 5.47-fold at 0.6 mM FA (**Figures 3A, B**). In contrast, 5-F-THF was a substantially stronger activator of NiR. Compared with the blank control, NiR activity increased approximately 62.38-fold at 1.0 mmol·L^–1^ 5-F-THF. Even at 0.1 mM, 5-F-THF significantly increased NiR activity relative to the control, with activity increasing progressively as the concentration increased. By comparison, FA increased NiR activity by only 8.49-fold at the corresponding concentration (**Figures 3C, D**). GS showed a much weaker response to both compounds. Neither compound markedly affected GS activity at low concentrations; only at 1 mM did FA and 5-F-THF slightly increase GS activity, to approximately 1.16- and 1.17-fold that of the control, respectively (**Figures 3E, F**). Both compounds also enhanced the activities of the GOGAT isozymes Fd-GOGAT and NADH-GOGAT, although their concentration responses differed. FA and 5-F-THF showed similar effects on Fd-GOGAT, with detectable stimulation at 0.1 mM that became stronger at higher concentrations (**Figures 3G, H**). For NADH-GOGAT, FA increased enzyme activity at concentrations as low as 0.2 mM, whereas 5-F-THF required a concentration of at least 0.5 mM to produce a measurable increase (**Figures 3I, J**). Overall, these results demonstrate that folate compounds can directly modulate key enzymes involved in nitrogen assimilation pathway, with the magnitude and concentration dependence of their effects differing substantially among enzymatic steps.

### 3.4 Exogenous FA application enhances nitrogen metabolism *in vivo* and reshapes metabolite profiles

#### 3.4.1 FA alters NR, NiR, GS, and GOGAT activities *in vivo*

To determine whether the effects observed *in vitro* also occurred in intact plants, two-year-old *D. officinale* plants were treated with foliar applications of FA at different concentrations. The *in vivo* responses were broadly consistent with the *in vitro* findings. Exogenous FA significantly increased leaf NR and NiR activities. At 15 mg·L^-1^, NR and NiR activities increased to 1.22- and 1.46-fold those of the control, respectively, indicating that foliar-applied FA can be absorbed and influence nitrogen metabolism *in planta* (**Figure 4A**; **Supplementary Figures 1A**, **B**). GS and NADH-GOGAT showed concentration-dependent responses, whereas Fd-GOGAT responded only modestly *in vivo*. GS activity increased by 11% at 5 mg·L^–1^ FA but did not differ significantly from the control at 15 mg·L^–1^ (**Figure 4A**; **Supplementary Figure 1C**). At 15 mg·L^–1^, Fd-GOGAT activity increased by only 6 % (**Figure 3A**; **Supplementary Figures 1D**), whereas NADH-GOGAT activity increased significantly to 1.22-fold that of the control (**Figure 4A**; **Supplementary Figure 1E**). Thus, FA promoted nitrogen metabolism in intact plants, although the magnitude and direction of the response varied among enzymes and depended on FA concentration.

**Figure 4:**
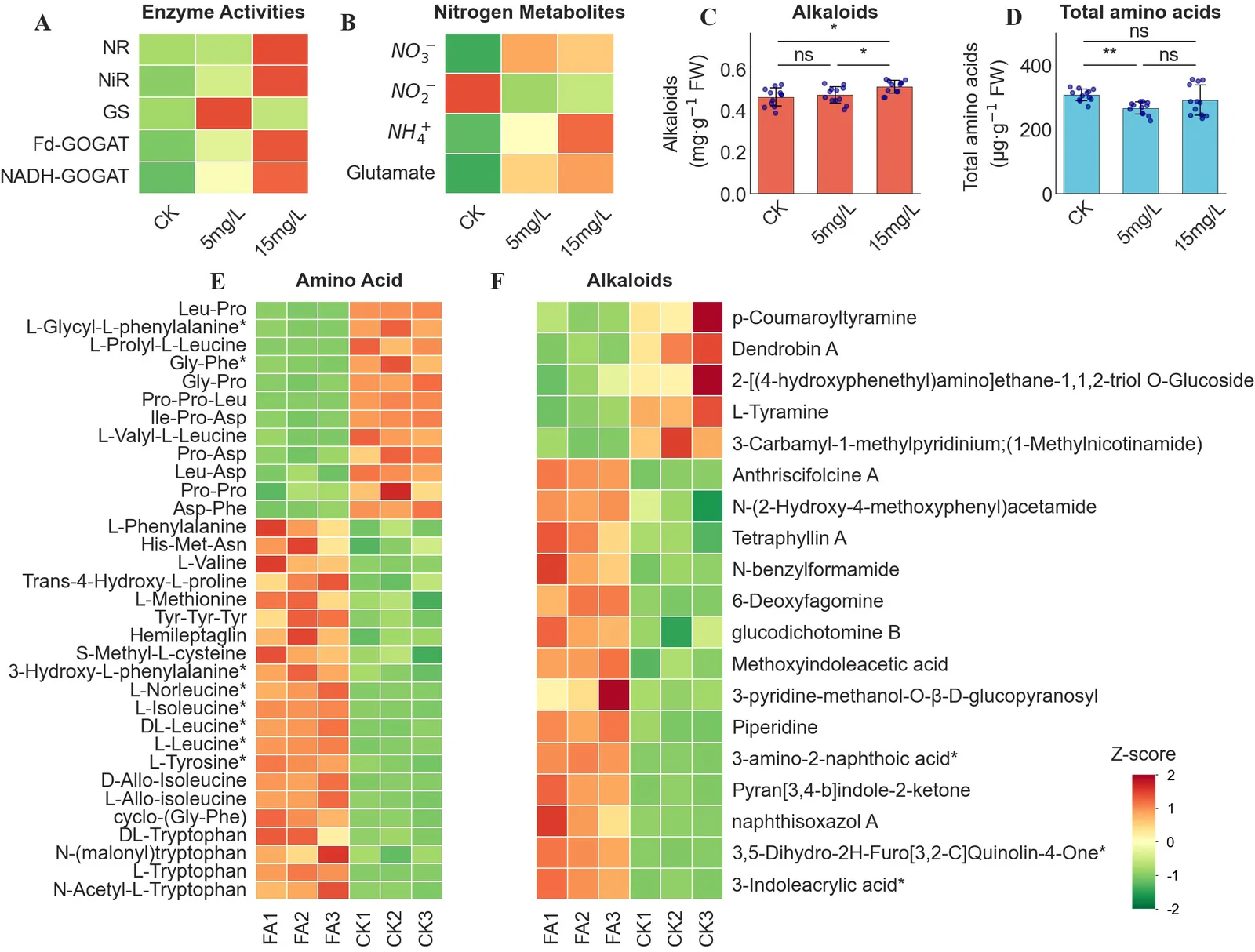
Folic acid (FA) spraying modulates nitrogen metabolism enzyme activities and related metabolite accumulation in *D. officinale in vivo*. **(A)** Heatmap of nitrogen-assimilating enzyme activities (NR, NiR, GS, Fd-GOGAT, and NADH-GOGAT) under different FA concentrations (CK, 5 mg·L^-1^ FA, 15 mg·L^-1^ FA). Data are presented as Z-scores calculated across treatment groups. **(B)** Heatmap of key nitrogen metabolites (NO_3_^−^, NO_2_^−^, NH_4_^+^, and Glu) under the same treatments. **(C, D)** Bar charts of total alkaloids **(C)** and total amino acids **(D)**. **(E)** Heatmap of 33 differentially accumulated amino acids and their derivatives, normalized to Z-scores, across control (CK1–CK3) and FA-treated (FA1–FA3) groups. **(F)** Heatmap of 19 differentially accumulated alkaloids, normalized to Z-scores, across control (CK1–CK3) and FA-treated (FA1–FA3) groups. Data in panels C and D are presented as mean ± SD (n = 12). Significant differences between treatments are indicated by P < 0.05, *P < 0.01, **P < 0.001, ***P < 0.0001; ns, not significant, as determined by Tukey’s multiple-comparison test.

#### 3.4.2 FA modulates inorganic nitrogen pools and Glu accumulation

To further assess the effects of FA on nitrogen metabolism, we measured nitrogen-related metabolites in *D. officinale* leaves following foliar FA application. At the inorganic nitrogen level, treatment with 5 and 15 mg·L^-1^ FA significantly increased nitrate content to 1.46- and 1.49-fold that of the control, respectively, while reducing nitrite content to 59% and 64% of the control, respectively (**Figure 4B**; **Supplementary Figures 1F**, **G**). Treatment with 15 mg·L^-1^ FA also increased ammonium content slightly, by 2% relative to the control (**Figure 4B**; **Supplementary Figure 1H**). Among the primary products of nitrogen assimilation, Glu content increased in a concentration-dependent manner, with 5 and 15 mg·L^-1^ FA increasing Glu levels by 17% and 28%, respectively, relative to the control (**Figure 4B**; **Supplementary Figure 1I**). Thus, FA altered inorganic nitrogen pools and promoted Glu accumulation, indicating enhanced nitrogen assimilation.

#### 3.4.3 FA remodels amino acid and alkaloid profiles

FA also markedly affected nitrogen-containing secondary metabolites. Alkaloid content did not differ significantly from the control at 5 mg·L^-1^ FA but increased markedly at 15 mg·L^–1^ (**Figure 4C**; **Supplementary Figure 1G**). In contrast, 5 mg·L^-1^ FA reduced total amino acid and total protein contents to 86% and 73% of the control, respectively, whereas 15 mg·L^–1^ FA restored both parameters to levels that were not significantly different from the control (**Figure 4D**; **Supplementary Figures 1K**, **L**).

Metabolomic analysis identified 33 differentially accumulated amino acids and derivatives between the control and FA-treated groups, including 21 upregulated and 12 downregulated metabolites (**Figure 4E**). The most strongly decreased metabolites were Leu-Pro, L-Glycyl-L-phenylalanine, L-Prolyl-L-leucine, and Gly-Phe, each of which decreased to < 10% of the control level. Conversely, L-phenylalanine and L-tyrosine increased to 2.05- and 3.36-fold those of the control, respectively, while L-methionine increased 2.55-fold. Notably, tryptophan and its derivatives showed the strongest accumulation, with L-tryptophan and N-acetyl-L-tryptophan reaching 5.21- and 10.60-fold the control levels, respectively.

Metabolomic analysis identified 19 differentially accumulated alkaloids in the FA-treated group, including 14 upregulated and 5 downregulated compounds (**Figure 4F**). p-Coumaroyltyramine and Dendrobin A decreased by 60% relative to the controls, whereas piperidine, naphthisoxazol A, 3,5-dihydro-2H-furo[3,2-c]quinolin-4-one, and 3-indoleacrylic acid increased to 3.47-, 3.81-, 4.08-, and 4.71-fold the control levels, respectively. These findings indicate that FA treatment substantially altered amino acid and alkaloid metabolic profiles in *D. officinale* leaves.

### 3.5 Molecular docking reveals direct interactions between folates and nitrogen metabolism enzymes

To investigate the potential molecular basis of folate-mediated regulation of nitrogen metabolism, molecular docking simulations were performed using AutoDock Vina. FA and 5-F-THF were docked against five key enzymes involved in nitrogen metabolism: NR, NiR, GS, Fd-GOGAT, and NADH-GOGAT. Because experimentally determined three-dimensional structures of these *D. officinale* enzymes are unavailable, the corresponding *A. thaliana* structures were used as templates. The identified *D. officinale* orthologs shared 62–84% pairwise sequence identity with the respective templates across the aligned regions (**Supplementary Table 1**), supporting their use as structural surrogates. Ramachandran plot analysis further showed that >90% of residues were located in the most favorable or additionally allowed regions (**Supplementary Figure 2**), indicating acceptable structural quality.

Both FA and 5-F-THF showed strong predicted binding to all five enzymes, with binding energies ranging from −8.145 to −9.673 kcal·mol^-1^. FA exhibited the strongest predicted binding to NiR (−9.673 kcal·mol^-1^), followed by NR (−9.597 kcal·mol^-1^), GS (−9.490 kcal·mol^-1^), and NADH-GOGAT (−9.456 kcal·mol^-1^). For 5-F-THF, the lowest binding energies were observed for NiR (−9.307 kcal·mol^-1^) and NADH-GOGAT (−9.264 kcal·mol^-1^), while the binding energies for NR, GS, and Fd-GOGAT were −8.582, −8.326, and −8.145 kcal·mol^-1^, respectively (**Table 1**). FA exhibited slightly lower predicted binding energies than 5-F-THF for all five enzymes. However, docking scores did not fully correspond to the enzyme activity patterns observed *in vitro*, particularly for NiR, for which 5-F-THF produced a stronger activity enhancement than FA.

**Table 1:** Docking scores of 5-F-THF and FA with key nitrogen metabolism enzymes (kcal·mol^-1^).

Compound	NR	NiR	GS	Fd-GOGAT	NADH-GOGAT
5-F-THF	-8.582	-9.307	-8.326	-8.145	-9.264
FA	-9.597	-9.673	-9.49	-9.225	-9.456

Structural analyses using PyMOL 3.0.3 and Discovery Studio showed that both compounds occupied binding pockets within the nitrogen-metabolism enzymes and formed multiple interactions, including hydrogen bonds, π-type interactions, and hydrophobic contacts (**Figure 5**; **Supplementary Figure 3**). For example, FA binding to NiR involved π-cation, π-sulfur, and π-alkyl interactions between the pteridine ring and residues within the binding pocket, whereas the Glu tail formed several conventional hydrogen bonds with polar residues, including Thr, Gln, Arg, and Lys (**Figure 5**). Similarly, 5-F-THF formed hydrogen-bond networks and hydrophobic interactions with NADH-GOGAT (**Supplementary Figure 3**). These docking results suggest that FA and 5-F-THF can directly interact with nitrogen-metabolism enzymes and may contribute to the changes in enzyme activity observed *in vitro*.

**Figure 5:**
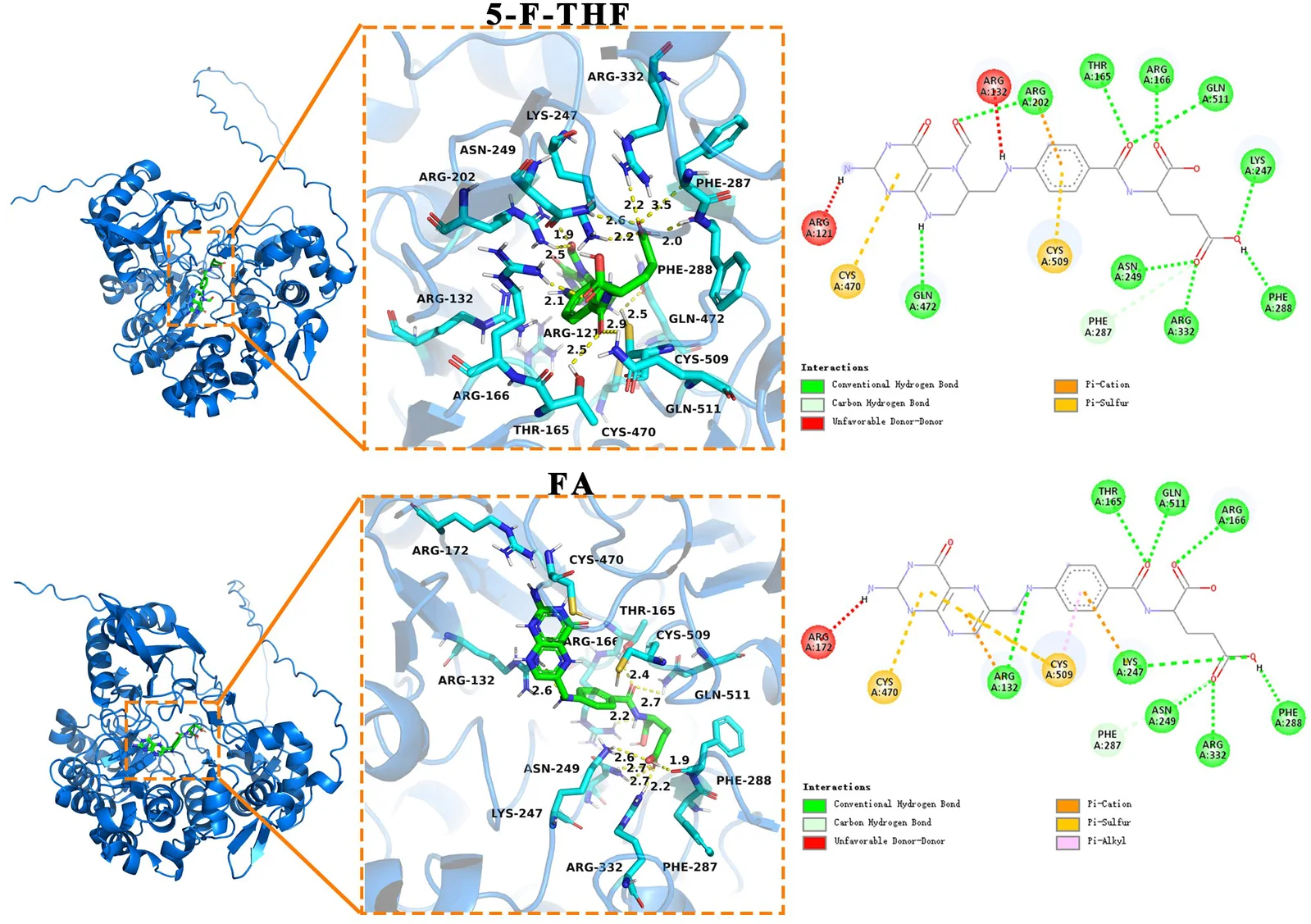
Molecular docking analysis of 5-formyltetrahydrofolate (5-F-THF) and folic acid (FA) with nitrite reductase (NiR). The left panel shows the overall docking conformation of the ligand–NiR complex, with NiR represented as a blue cartoon and the ligand highlighted in green. The middle panel shows a close-up of the binding pocket, including key amino acid residues within 3.5 Å of the ligand and the corresponding interaction distances (Å). The right panel shows a 2D schematic of ligand–protein interactions; green dashed lines represent conventional hydrogen bonds, orange dashed lines π-cation interactions, yellow dashed lines π-sulfur interactions, pink dashed lines π-alkyl interactions, and red dashed lines unfavorable donor–donor interactions.

## 4 Discussion

### 4.1 Tissue-specific folate accumulation patterns and nutritional regulation in *D. officinale*

This study systematically characterized the distribution of folate derivatives across tissues and developmental stages in *D. officinale*. Leaves accumulated the highest levels of M-glu1 and M-glu2, whereas flowers preferentially accumulated formylated folates (10-F-glu1 and 5-F-glu1). In contrast, folate contents in stems declined with plant age (**Figure 1**). This tissue-specific distribution is consistent with the distinct physiological functions of these organs. As major sites of photorespiration and amino acid metabolism, leaves require substantial folate pools to support one-carbon metabolism (Meng et al., 2025b; Hankofer et al., 2026). As reproductive tissues, flowers may have greater demands for formylated folates to support nucleotide biosynthesis and rapid cell proliferation (Wang et al., 2017). The age-dependent decline in stem folate content may reflect reduced metabolic activity in mature stem tissues (Yuan et al., 2019; Wu et al., 2023b).

Compared with model species such as *Arabidopsis* and maize (Li et al., 2021; Meng et al., 2025b), *D. officinale* exhibited a more pronounced tissue-specific folate distribution pattern. This difference may be associated with its epiphytic growth habit, which exposes the plant to fluctuating nutrient availability and may favor more specialized metabolic partitioning to improve resource-use efficiency. These findings provide further insight into folate accumulation patterns in *D. officinale*.

We further investigated the effects of three commercial foliar fertilizers on folate derivative profiles and total folate accumulation in *D. officinale* leaves (**Figure 2**). The balanced water-soluble fertilizer Huaduoduo No. 1 had the strongest overall effect, significantly increasing the contents of 10-F-glu1, M-glu1, and total folate (**Figures 2A, C, E**). This response may be attributable to its balanced nutrient supply, particularly nitrogen availability, which can support glutamate biosynthesis and folate polyglutamylation (Gorelova et al., 2019). Balanced fertilization may therefore promote folate accumulation by coordinately enhancing upstream precursor supply and downstream one-carbon metabolism (Basset et al., 2005; Scaglione and Panzavolta, 2014; Gorelova et al., 2019).

The trace-element fertilizer ManCui significantly increased M-glu2 accumulation (**Figure 2D**), suggesting that micronutrients such as zinc and iron may contribute to folate metabolism. These elements serve as cofactors for several enzymes involved in folate-related pathways and may consequently influence the accumulation of specific folate derivatives (Basset et al., 2005; Lian et al., 2015; Gorelova et al., 2019). In addition, the high-potassium formulation ManCui 20-10-20 + 0.5Ca+1Mg+TE preferentially increased 5-F-glu1 content (**Figure 2B**), indicating that fertilizer composition may differentially influence distinct branches of folate metabolism.

Combined treatment with exogenous FA and foliar fertilizers did not produce a synergistic effect and, in some cases, reduced folate derivative contents. FA alone had limited effects on most folate-related indicators in *D. officinale* (**Figure 2**). One possible explanation is the tight feedback regulation of folate metabolism. As an oxidized folate form, exogenous FA must first be reduced to THF before entering the endogenous one-carbon metabolic network, which may limit its effective utilization (Scaglione and Panzavolta, 2014). In addition, increased THF levels may exert feedback inhibition on *de novo* folate biosynthesis (Basset et al., 2005; Bekaert et al., 2008). Chemical interactions between FA and mineral nutrients, together with competition for foliar uptake, may further reduce the bioavailability of both components (Alshaal and El-Ramady, 2017; Strobbe and Van Der Straeten, 2017). These findings suggest that effective folate biofortification in *D. officinale* may require coordinated regulation of nutrient supply and endogenous folate metabolism rather than simple supplementation with folate precursors.

Folate also showed pronounced tissue-specific distribution across organs of *D. officinale*. Accumulating evidence indicates that nitrogen metabolites and the activities of nitrogen-metabolizing enzymes likewise exhibit tissue-preferential distributions, consistent with the larger folate pool observed in leaves in the present study. Physiological and transcriptomic studies have shown that NR and NiR activities are significantly higher in leaves than in roots (Yoneyama and Suzuki, 2019; Han et al., 2022). This organ-level differentiation in nitrogen metabolism may provide a physiological basis for the tissue-specific coordination of folate distribution and nitrogen assimilation in *D. officinale*.

### 4.2 Direct activation of nitrogen metabolism enzymes by folate compounds

5-F-THF and FA directly activated key nitrogen-metabolism enzymes in *D. officinale*, including NR, NiR, GS, and GOGAT. Together with the *in vivo* validation, metabolite analyses, and molecular docking results, these findings support a close association between folate derivatives and nitrogen metabolism regulation in this species.

Folates are traditionally recognized as carriers of one-carbon units in one-carbon metabolism (Li et al., 2025). Our findings suggest that folate derivatives may also regulate nitrogen metabolism beyond their classical cofactor function. This interpretation is consistent with previous evidence that 5-F-THF can bind to folate-related and nitrogen metabolism-associated proteins in *Arabidopsis* (Li et al., 2021) and that folate metabolism perturbation can induce nitrogen-starvation-like responses (Meng et al., 2025b). Recent studies have further highlighted potential signaling roles for B vitamins in plant development and stress responses (Fitzpatrick, 2024). Our results extend this emerging view by providing additional evidence that folate derivatives may directly modulate nitrogen assimilation processes in plants.

Among the tested enzymes, NiR showed the strongest response to 5-F-THF, with a 62.38-fold increase in activity, substantially greater than that induced by FA (**Figure 5**). NiR catalyzes the reduction of nitrite to ammonium and is therefore central to nitrogen assimilation (Yu et al., 2021). The stronger activation by 5-F-THF may reflect differences in folate structure and protein interactions. Unlike FA, 5-F-THF is a reduced folate containing a formyl group, which may promote more favorable interactions within the NiR protein environment (Yamada et al., 2023). Because NiR activity also depends on redox state and electron transfer, these properties may contribute to its sensitivity to 5-F-THF (Yamada et al., 2023).

Molecular docking showed that both FA and 5-F-THF could bind the active pockets of nitrogen-metabolism enzymes, although FA exhibited slightly lower binding energies than 5-F-THF. Notably, stronger predicted binding did not necessarily result in greater enzyme activation, indicating that binding strength alone cannot explain the observed regulatory effects. Differences in binding position, interaction networks, and ligand-induced conformational changes may instead contribute to the distinct effects of FA and 5-F-THF (Roskoski, 2021; He et al., 2022). Such discrepancies between docking scores and enzymatic responses are common in ligand–protein systems and highlight the need to consider both binding properties and functional effects.

The *in vivo* experiments further showed that folate treatment increased NR and NiR activities and altered nitrogen metabolite levels, including increased nitrate accumulation and reduced nitrite content. These changes are consistent with enhanced nitrogen assimilation and support the physiological relevance of the *in vitro* enzyme activation results. Thus, folate compounds can influence nitrogen metabolism in *D. officinale* at both enzymatic and metabolic levels.

### 4.3 A working model for folate-nitrogen metabolic crosstalk

Together with previous studies, our results support a working model in which folate compounds regulate nitrogen metabolism in *D. officinale* through multiple interconnected mechanisms. 5-F-THF and FA may directly modulate nitrogen-metabolism enzymes through specific molecular interactions, including hydrogen bonds, π-type interactions, and hydrophobic contacts. Such regulation could provide a rapid response mechanism independent of transcriptional reprogramming and may therefore be particularly relevant under fluctuating environmental or nutritional conditions.

Folate-mediated one-carbon metabolism is closely interconnected with nitrogen metabolism at multiple metabolic nodes. In plants, serine hydroxymethyltransferase and the glycine decarboxylase complex link photorespiration with one-carbon metabolism, thereby coupling carbon, nitrogen, and photorespiratory fluxes (Duan et al., 2022). Recent studies further indicate that the serine–glycine–one-carbon network coordinates nitrogen and sulfur metabolism and contributes to developmental regulation (Rosa-Téllez et al., 2024). Formate-tetrahydrofolate ligase can also supply one-carbon units from glycolate to the cytosolic one-carbon network (Saeheng et al., 2024), providing an additional link among folate, carbon, and nitrogen metabolism.

Folate metabolism is itself subject to feedback regulation. Previous studies have shown that 5-F-THF inhibits key enzymes involved in folate biosynthesis (Li et al., 2021), suggesting homeostatic regulation of folate pool size. In this context, the activation of nitrogen metabolism enzymes observed here may form part of a broader feedback loop linking folate status, nitrogen assimilation, amino acid synthesis, and one-carbon unit supply, thereby coordinating folate metabolism with nitrogen utilization.

Folate–nitrogen crosstalk may also extend to signaling networks. NR has been reported to contribute to nitric oxide (NO) production under certain conditions (Khan et al., 2023), suggesting that folate-mediated modulation of NR could influence NO signaling. Given the interplay among NO, reactive oxygen species, and plant hormones, folate-regulated nitrogen metabolism may consequently affect stress responses and developmental processes.

### 4.4 Exogenous folic acid remodels nitrogen and secondary metabolism in *D. officinale*

Our results indicate that exogenous FA broadly regulates the metabolic network of *D. officinale*, from inorganic nitrogen assimilation (**Figure 4B**) to amino acid metabolism (**Figure 4E**) and downstream secondary metabolite accumulation (**Figure 4F**). Thus, FA appears to promote coordinated metabolic reprogramming rather than acting on a single pathway.

Regarding inorganic nitrogen metabolism, FA treatment increased nitrate but decreased nitrite levels in leaves (**Figure 3B**; **Supplementary Figures 1F**, **G**). This pattern is consistent with our *in vitro* results showing that FA and 5-F-THF directly activate NR and NiR (**Figures 3A–D**). The decrease in nitrite is consistent with enhanced NiR activity, whereas the increase in nitrate may reflect stimulated nitrate uptake or transport. FA also increased ammonium levels and promoted Glu accumulation in a concentration-dependent manner (**Supplementary Figures 1H**, **I**), consistent with the observed activation of GS and GOGAT *in vitro* (**Figures 3E, H, J**). Together, these results suggest that FA enhances nitrogen assimilation and promotes the conversion of inorganic nitrogen into organic forms. This interpretation is further supported by our previous work in *Arabidopsis atdfb* mutants, in which disrupted folate metabolism impaired nitrogen uptake and assimilation (Meng et al., 2021).

At the amino acid level, FA produced a dose-dependent effect on total amino acid and protein contents. Low-dose FA reduced both parameters, whereas higher concentrations restored them to levels comparable to the control (**Figure 3D**; **Supplementary Figure 1K**), indicating a non-linear response to folate supply. One possible explanation is that folate stimulation of one-carbon metabolism alters the balance between amino acid utilization and biosynthesis (Danchin et al., 2020). Increased folate availability may also improve nitrogen assimilation, compensating for amino acid consumption and restoring steady-state pools. FA also substantially altered individual amino acids, with aromatic amino acids, including phenylalanine, tyrosine, and tryptophan, and methionine showing marked accumulation (**Figure 3E**). This selective enrichment suggests that FA reshapes specific metabolic branches rather than globally enhancing amino acid metabolism. Methionine accumulation may reflect increased one-carbon flux and SAM-dependent methylation capacity (González and Vera, 2019; Watanabe et al., 2021; Escaray et al., 2022), whereas aromatic amino acid enrichment may increase precursor availability for phenolic, flavonoid, and alkaloid biosynthesis (Maeda and Dudareva, 2012; Parthasarathy et al., 2018).

Consistent with this interpretation, FA substantially altered the alkaloid profile, with 19 alkaloids differentially accumulated and most showing increased abundance. Notably, piperidine-, indole-, and other alkaloid-related compounds were particularly enriched (**Figure 3F**). These changes may reflect increased precursor availability through amino acid metabolism and enhanced folate-dependent one-carbon supply for methylation reactions involved in alkaloid biosynthesis (González and Vera, 2019; Escaray et al., 2022). Previous studies have shown that folate-associated genes are co-expressed with amino acid metabolism and phenylpropanoid biosynthesis-related genes (Song et al., 2021), supporting a role for folate status in regulating secondary metabolism through coordinated metabolic networks (Pott et al., 2019).

Together, these data support a model in which exogenous FA drives metabolic reprogramming in *D. officinale* by linking inorganic nitrogen assimilation, amino acid remodeling, and secondary metabolite production. Rather than uniformly activating metabolic pathways, folate appears to promote selective allocation of carbon and nitrogen toward specific branches, particularly aromatic amino acid and alkaloid biosynthesis. This metabolic reallocation may help maintain metabolic balance while enhancing the production of bioactive compounds (Jan et al., 2021; Wu et al., 2024).

### 4.5 Practical implications and future directions for folate biofortification in *D. officinale*

This study provides a basis for folate biofortification in *D. officinale* by revealing tissue-specific folate accumulation patterns and a functional link between folate metabolism and nitrogen assimilation. Our findings suggest that folate derivatives may function not only as metabolic cofactors but also as regulatory molecules that modulate key nitrogen assimilation enzymes, expanding current understanding of folate function in medicinal plants and informing future quality improvement strategies.

From a practical perspective, several approaches may improve the nutritional quality of *D. officinale*. Foliar fertilization modulated folate accumulation in a formulation-dependent manner, with the balanced nutrient solution producing the most favorable effect, suggesting that optimized nutrient balance may be more effective than single-component supplementation. Tissue-specific folate distribution also supports targeted metabolic engineering, whereby folate biosynthesis or transport could be enhanced in specific organs to promote accumulation of desired folate forms in edible or medicinal tissues. Exogenous FA application may additionally redirect metabolic flux, although the dose-dependent responses observed here highlight the need for careful concentration control. Low-dose FA favored alkaloid accumulation, whereas higher doses were associated with restoration of amino acid and protein levels.

Several limitations warrant consideration. Molecular docking was based on *Arabidopsis* protein structures because corresponding *D. officinale* structures are unavailable, although the relevant proteins share high sequence similarity. Physiological validation was also conducted under greenhouse conditions; whether these effects persist under field cultivation remains unknown. Future studies should therefore validate these findings in field trials and obtain structural evidence, such as crystallographic or cryo-EM data, to define the binding sites and conformational changes underlying folate-mediated enzyme activation. This study provides a framework for understanding folate-nitrogen metabolic crosstalk in *D. officinale* and offers practical insights into folate biofortification and medicinal quality improvement.

## 5 Conclusion

Overall, this study proposes that folate compounds act as regulatory mediators linking nitrogen assimilation with primary and secondary metabolism in *D. officinale*. Tissue-specific profiling revealed distinct folate distribution patterns, with leaves enriched in total folates and M-type reduced folates, whereas flowers preferentially accumulated formylated folates. *In vitro* enzyme assays showed that both 5-F-THF and FA directly activate key nitrogen assimilation enzymes, with 5-F-THF increasing NiR activity by 62.38-fold. Exogenous FA application further stimulated nitrogen assimilation and altered amino acid and alkaloid profiles *in vivo*. Molecular docking supported direct interactions between folates and the target enzymes, with binding energies of −8.1 to −9.7 kcal·mol^-1^. Collectively, these findings provide mechanistic insight into folate-nitrogen crosstalk and highlight folate-oriented metabolic manipulation as a potential strategy for enhancing the nutritional and medicinal value of *D. officinale*.

## Data Availability

The datasets presented in this study can be found in online repositories. The names of the repository/repositories and accession number(s) can be found in the article/**Supplementary Material**.
