# Supplementary material for: Foliar folic acid application enhances nitrogen assimilation and improves quality-related metabolites accumulation in Dendrobium officinale
Source: Front Plant Sci. 2026 Sep 14;17:1918138. doi: 10.3389/fpls.2026.1918138 (PMC13618328; doi:10.3389/fpls.2026.1918138)
Supplement: Supplementary file 1 [file DataSheet1.docx]

**Figure S1. Folic acid (FA) spraying modulates nitrogen metabolism enzyme activities and related metabolite accumulation in *Dendrobium officinale* *in vivo*.** (A–E) Activities of nitrate reductase (NR, A), nitrite reductase (NiR, B), glutamine synthetase (GS, C), ferredoxin-dependent glutamate synthase (Fd-GOGAT, D), and NADH-dependent glutamate synthase (NADH-GOGAT, E) under different treatments (CK, 5 mg·L^-1^ FA, and 15 mg·L^-1^ FA). (F–L) Contents of key nitrogen metabolites: nitrate (NO₃⁻, F), nitrite (NO₂⁻, G), ammonium (NH₄⁺, H), glutamate (I), total alkaloids (J), total amino acids (K), and total protein (L). Data are presented as mean ± SD (n = 12). Different lowercase letters above bars indicate significant differences among treatments at P < 0.05, as determined by Tukey’s multiple-comparison test.

**Figure S2. Ramachandran plot validation of the five nitrogen metabolism enzymes used for molecular docking.** Ramachandran plots show the distribution of backbone dihedral angles (φ, ψ) for the modeled enzyme structures: nitrate reductase (NR), nitrite reductase (NiR), glutamine synthetase (GS), ferredoxin-dependent glutamate synthase (Fd-GOGAT), and NADH-dependent glutamate synthase (NADH-GOGAT). The plots are divided into four regions: most favored (red), additionally allowed (yellow), generously allowed (light yellow), and disallowed (white). Blue dots represent individual amino acid residues. The statistics below each plot indicate the percentage of residues in each category. All structures had >90% of residues in the most favored and additionally allowed regions, supporting the quality of the structural models used for docking simulations.

**Figure S3. Molecular docking analysis of 5-formyltetrahydrofolate (5-F-THF) and folic acid (FA) with five nitrogen metabolism enzymes.** For each panel, the left part shows the overall docking conformation of the ligand–enzyme complex, with the enzyme represented as a blue cartoon and the ligand highlighted in green. The right part shows a close-up of the binding pocket, including key amino acid residues within 3.5 Å of the ligand and the corresponding interaction distances (Å).
